# Applying the Theoretical Domains Framework to understand knowledge broker decisions in selecting evidence for knowledge translation in low- and middle-income countries

**DOI:** 10.1186/s12961-019-0463-9

**Published:** 2019-06-11

**Authors:** Theresa C. Norton, Daniela C. Rodriguez, Sara Willems

**Affiliations:** 1 Jhpiego, 1615 Thames Street, Baltimore, MD 21231 United States of America; 2 Department of International Health, Johns Hopkins Bloomberg School of Public Health, 615 N. Wolfe Street, Baltimore, MD 21205 United States of America; 3 Department of Public Health and Primary Care, Ghent University, Campus UZ, K3, 6de verdieping, Corneel Heymanslaan 10, 9000 Ghent, Belgium

**Keywords:** Knowledge brokers, knowledge translation, theoretical domains framework, barriers, facilitators, low- and middle-income countries

## Abstract

**Background:**

Health-related organisations disseminate an abundance of clinical and implementation evidence that has potential to improve health outcomes in low- and middle-income countries (LMICs), but little is known about what influences a user decision to select particular evidence for action. Knowledge brokers (KBs) play a part as intermediaries supporting evidence-informed health policy and practice by selecting and synthesising evidence for research users, and therefore understanding the basis for KB decisions, can help inform knowledge translation strategies. The Theoretical Domains Framework (TDF), a synthesis of psychological theories, was selected as a promising analysis approach because of its widespread use in identifying influences on decisions to act on evidence-based healthcare guidelines. This study explored its application in the context of KB decisions regarding evidence for use in LMICs.

**Methods:**

The study analysed data collected from participants of a 2015 global maternal and newborn health conference in Mexico. A total of 324 conference participants from 56 countries completed an online survey and 20 from 15 countries were interviewed about evidence use and sharing after the conference. TDF domains and constructs were retrospectively applied and adapted during coding of qualitative data to enhance understanding of the KB decision process in selecting evidence for action.

**Results:**

Application of the TDF involved challenges related to overlapping constructs, retrospective use, and complexities of global health settings and relevant knowledge. Codes needed to be added or adapted to account for how KBs’ internal reflections on external factors influenced their actions in selecting evidence to share and use, and the decisions they made during the process. Four themes of the rationale for changing the TDF were identified during analysis, namely Influences from Beyond the Organisation, Knowledge Selection as a Process, Access and Packaging of Knowledge, and Fit for Use.

**Conclusions:**

Theories of individual behaviour, such as those in the TDF, can enhance understanding of the decisions made by actors such as KBs along dissemination and knowledge translation pathways. Understanding how KBs reflect on evidence and interact with their environment has the potential for improving global dissemination efforts and LMIC-to-LMIC exchange of implementation evidence.

**Electronic supplementary material:**

The online version of this article (10.1186/s12961-019-0463-9) contains supplementary material, which is available to authorized users.

## Background

Determining the factors that influence successful dissemination and uptake of evidence-based, context-appropriate health practices has critical importance for reducing maternal and newborn mortality in low- and-middle income countries (LMICs), which carry the greatest burden of preventable deaths [1, 2]. The how and why of research uptake – a topic labelled as knowledge translation (KT) in the literature – has been widely studied to address the long-standing gap between health research evidence and practice [3]. The methods associated with health KT include filtering and packaging evidence to suit the needs of health system audiences, disseminating the knowledge and advocating for its application in decision-making [3–5]. Global commitment in the form of actions and communications from organisations such as WHO and PAHO, and multi-region programmes such as the Evidence into Policy Networks [5], point to the importance of KT.

Despite keen interest by the health research community and global commitment, no one standard for KT emerges as widely accepted among the many proposed theories and frameworks [4–8]. Among the array of KT explanations and predictions, many focus broadly on systems, infrastructure and activities supporting KT, whilst others focus more narrowly on individual decisions to adhere to a specific evidence-based clinical care guideline [4, 6]. A common element among the perspectives on KT (whether explicitly stated or not) is the individual health system actor who makes decisions after learning about evidence within a larger context.

The way in which literature addresses critical elements of KT suggests the importance of individual perspective and choices about evidence. While dissemination of knowledge is a crucial aspect of KT, publication alone without interpersonal communication has been shown to be less effective in promoting evidence-informed decision-making [7, 9]. Individual behaviour comes into play during KT when choosing knowledge to package for audiences [5], cultivating relationships between researchers and decision-makers to influence research agendas and uptake [7], and interacting with patients to provide high-quality, evidence-based care [10]. Understanding how individuals perceive their context and settings can reveal barriers and facilitators to changing behaviour regarding evidence use [11]. Despite the importance of individual actors in KT, current KT theories and frameworks focus less on their decision processes and influences of internal and external factors in making choices when faced with an abundance of evidence.

In the context of public health, individual or organisation knowledge brokers (KBs) serve as intermediaries between research producers and consumers to facilitate KT – production and context-appropriate use of evidence to inform decision-making in health policy and practice [12–14]. KBs synthesise and disseminate evidence to support health policy, practice or clinical reasoning when and where the knowledge is needed. Through active relationships, KBs address the near-term needs of decision-makers by curating knowledge that is most applicable and communicating it in terms understandable to the decision-maker or other knowledge users. Understanding KB thought processes about selection and sharing of evidence has relevance, therefore, to strengthening evidence-informed decision-making.

Use of psychological theory to understand individual decisions about evidence – such as those facing KBs – has origins in studies on internal and external factors influencing use of social science research in the 1970s [15] and applies to current KT interventions [10]. As calls have increased for the use of theory in designing KT interventions as a way to improve results, implementation researchers have increasingly adopted a consolidated theoretical approach called the Theoretical Domains Framework (TDF). Recognising that no one theory is sufficient to address the complexities of behaviour in healthcare settings, the TDF consolidates aspects of 33 theories into a framework of 14 theoretical domains with component constructs in the validated version used in this study [16]. The domains, which are listed in Box 1, include internally reflecting concepts, such as Beliefs about Capabilities, and externally oriented concepts, such as Environmental Context and Resources. Constructs provide details about the topics included within each domain (e.g., Fear and Anxiety within the domain Emotion). The TDF has been used extensively to identify barriers and facilitators for individual uptake of evidence-based practices, and more broadly for implementation design and research such as that embedded in the comprehensive Tailored Implementation for Chronic Diseases Checklist [17].

Whether used alone or with other frameworks, the TDF has been shown to be useful by offering a broad range of constructs that may influence individual decisions to make use of evidence [3, 18].

While the complexity of healthcare settings and associated decision-making is widely accepted, such complexity is more significant in global health, where wide variations in provider roles, culture and socioeconomic factors [19] provide challenges to understanding behaviour. Surprisingly, despite the pressing need for evidence-informed decision-making in health policy and practice in LMICs, few studies in those contexts have used the TDF. Where studies in LMICs have used the TDF, they have mostly focused on clinical behaviour such as guidelines implementation in Kenyan hospitals [20] and medication safety in Ethiopian hospitals [21]. Researchers have made little, if any, use of the TDF to understand the behaviour of health system actors in various roles related to uptake of research evidence. This paper describes application and adaptation of the TDF to explore KT in LMICs by better understanding internal and external barriers and facilitators facing KBs.

### Study context

The context of the study was the Global Maternal Newborn Health 2015 Conference held in Mexico City, October 18–21, 2015. Multiple organisations and programmes working globally to improve maternal and newborn health (e.g. Saving Newborn Lives at Save the Children) collaborated in convening the conference. Conference organisers designed the event for sharing evidence and planning future action in health research, policy and practice to improve health outcomes, particularly in LMICs [22]. To meet these goals, the conveners invited participants in a range of health system roles, such as researchers, policy-makers, funders and healthcare faculty members and providers. The outreach aimed to bring together health system actors who were anticipated to take later action, such as disseminating and discussing evidence with stakeholders to work towards better-informed health policy and practice. The invitation strategy suggests that organisers were targeting participants expected to act as KBs. Studies by the same researchers and also aiming to explore knowledge use and sharing after global maternal and newborn health conferences convened by some of the same organisers were conducted in 2012 and 2013 [23].

## Methods

### Target population

The target population for the study was participants in the 2015 Global Maternal Newborn Health Conference held in Mexico City in 2015.

### Study design

**Fig. 1:**
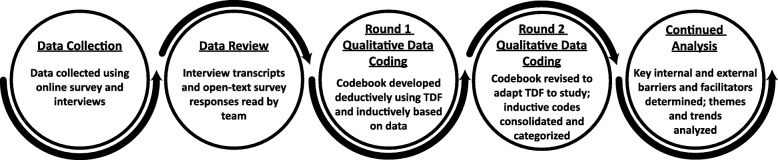
Overview of data collection and analysis process. Interview and survey data were iteratively analysed using TDF-derived codes, other codes from the literature and inductively derived codes

Authors used a mixed methods explanatory sequential design [24]. Quantitative survey data were collected to capture conference participant demographics and characteristics of post-conference knowledge sharing and use (e.g. with whom they shared). Quantitative measures were first used to determine whether or not knowledge sharing and use had occurred after the conference and their parameters. Qualitative interview data were collected to explain and understand the quantitative data, that is, why or why not and how knowledge sharing and use occurred. Authors triangulated data sources (i.e. surveys, interviews and conference documents) to inform further data collection and analysis and provide richer insights into knowledge sharing and use. For example, researchers would compare examples of evidence use provided in an open-text field of the survey with comparable examples given during interviews and descriptions of the evidence in the conference documentation. The TDF provided a framework for identifying influences on decisions to use and share knowledge. Figure 1 illustrates the data collection and analysis process.

### Study procedures

Quantitative data were collected using a self-administered online survey. Qualitative data were collected using three methods, namely (1) including open-ended questions in the survey (“Q18. Please give an example of how you have used information or knowledge from this conference, if applicable”); (2) conducting semi-structured interviews with selected survey respondents; and (3) reviewing documents related to the conference (e.g. session descriptions in the programme) to understand the context of respondents’ comments about particular knowledge they shared and used. Both the survey and interviews were conducted in English. Verbal translation was provided for one interview respondent who communicated through an interpreter.

#### Instruments

The 22-question survey was intended to determine if respondents shared knowledge from the conference and with whom, and if they used it and how they used it, among other subjects. The 15 interview questions were intended to obtain additional details about respondent experiences sharing and using knowledge from the conference and influences on their decisions regarding sharing and use (see Additional files 1 and 2 for data collection instruments).

Both the survey instrument and interview script were developed and validated during studies conducted by the researchers in 2012 and 2013 also aiming to explore knowledge use and sharing after global maternal and newborn health conferences [23]. For the 2015 study, minor refinements consisted of adding multiple choice options that previous respondents entered as ‘Other’ responses. Expert review by researchers and conference organisers validated the changes.

#### Recruitment

Respondents were recruited through an e-mail invitation to complete a survey that was sent to a communications distribution list used by conference organisers to reach potential attendees. A subset of the list had attended the conference (*n* = 1000 in-person participants; number of online participants unknown). The distribution list was used as a convenient way of reaching the target population since conference organisers did not maintain a database of contact information for actual conference attendees. The team sent the initial e-mail message 9 months after the conference with reminders sent 2 and 3 weeks later. The survey closed after 1 month.

The interview candidates were purposively selected from the pool of survey respondents. Among survey respondents, 124 (38.3%) answered ‘Yes’ to a question asking if they would be willing to participate in a 30-min interview about their knowledge sharing and use experience following the conference. From the pool, TN purposively selected a sample of 20 survey respondents to contact for semi-structured interviews based on maximum diversity of country, type of work, type of organisation, mode of participation (online versus in-person), abstract acceptance (Y/N), and use or sharing of knowledge from the conference (Y/N). The study budget and timeline determined the target number of interviews. The variables for participant diversity were selected with the rationale – based on the researchers’ 2012 and 2013 similar studies [23] – that they would result in capturing perspectives with a variety of motivations to share and use evidence and multiple contexts. After three attempts to contact a potential respondent with no response, the protocol called for ruling out the candidate. TN continued to replenish the pool whilst maintaining diversity. Each 30-min interview was conducted using Skype (www.skype.com) or phone and audio recorded with permission, then transcribed. The lead author and trained interns conducted the interviews in English. One interview respondent used an interpreter. Interviews were completed between 10 and 14 months after the conference.

#### Inclusion and exclusion criteria

Inclusion/exclusion criteria for survey respondents was based on their confirmation of attending the 2015 Global Maternal and Newborn Health Conference either in person or online. Inclusion/exclusion criteria for participation in the semi-structured interviews was entering a valid e-mail address in response to the survey question, “May we contact you for a 30-minute interview to hear more about your experience using and sharing information and knowledge from the conference?” Potential participants that supplied an e-mail address that bounced back as undeliverable or failure to respond to follow-up e-mails from the researchers also resulted in exclusion.

#### Consent

The study protocol was reviewed by the lead author’s institutional review board and was determined to be exempt from further review. Nevertheless, interviewers obtained and recorded verbal consent by signing and dating consent forms, which was done during study as an ethical practice. Consent was not obtained from respondents who answered the survey as responses were not stored with personally identifiable information. The e-mail invitation to complete the survey said that respondents to the survey could be entered into a prize drawing for a Kindle Fire. There was no incentive to participate in the interviews.

### Sample characteristics

The online survey received responses to some or all questions from 324 of 1000 in-person conference participants (a 32.4% response rate) and additional online participants. Respondents represented 57 countries. A total of 64 candidates were contacted to be interviewed, of which 20 respondents from 15 countries were successfully interviewed. Participant countries consisted of both LMICs and higher-income countries as organisers invited participants based on the relevance of their evidence or work to global issues of maternal and newborn health, not on the country in which they were based. The study participants, as with the conference invitees, included a variety of health system roles relevant to facilitating understanding, sharing and use of the evidence presented at the conference. These roles included researchers, policy-makers, funders, programme implementers and healthcare professionals.

Table 1 presents characteristics of survey respondents and the subset interviewed.

**Table 1:** Characteristics of survey participants and the subset interviewed

Characteristics	Survey respondents (*n* = 324)	Subset interviewed (*n* = 20)
Mode of attendance		
In-person	252 (77.8%)	17 (85.0%)
On-line	72 (22.2%)	3 (15.0%)
Region		
Africa	108 (33.3%)	6 (30.0%)
Americas	102 (31.5%)	3 (15.0%)
Asia	83 (25.6%)	9 (45.0%)
Europe	15 (4.6%)	2 (10.0%)
Oceania	5 (1.5%)	0 (0.0%)
Unknown	11 (3.5%)	0 (0.0%)
Type of organisation		
Academic/Research Institution	83 (25.6%)	4 (20.0%)
Consultant	4 (1.2%)	0 (0.0%)
Donor	14 (4.3%)	0 (0.0%)
FBO	2 (0.6%)	0 (0.0%)
Government/Ministry	39 (12.0%)	5 (25.0%)
Media	2 (0.6%)	1 (5.0%)
Medical/Health Organisation	32 (9.9%)	2 (10.0%)
NGO/PVO (Local and International)	118 (36.4%)	7 (35.0%)
Private Sector (For-Profit)	4 (1.2%)	1 (5.0%)
United Nations System	15 (4.6%)	0 (0.0%)
Unknown	11 (3.6%)	0 (0.0%)
Type of Work		
Advocacy	19 (5.9%)	2 (10.0%)
Combination	3 (0.9%)	0 (0.0%)
Health/Medical Service Delivery	36 (11.1%)	5 (25.0%)
Health Communication	14 (4.3%)	0 (0.0%)
Policy-making	12 (3.7%)	1 (5.0%)
Programme Development/Management/Implementation	131 (40.4%)	4 (20.0%)
Research/Evaluation	72 (22.2%)	6 (30.0%)
Student	5 (1.5%)	0 (0.0%)
Teaching/Training	20 (6.2%)	2 (10.0%)
Unknown	12 (3.8%)	0 (0.0%)
Years in profession		
0–5 years	72 (22.2%)	3 (15.0%)
6–10 years	86 (26.5%)	6 (30.0%)
11–15 years	70 (21.6%)	6 (30.0%)
16 or more years	85 (26.2%)	5 (25.0%)
Unknown	11 (3.6%)	0 (0.0%)
Abstract accepted to conference		
Do not know	4 (1.2%)	1 (5.0%)
No	149 (46.0%)	8 (40.0%)
Yes	140 (43.2%)	11 (55.0%)
Unknown	31 (9.6%)	0 (0.0%)

*FBO* faith-based organisation, *NGO* non-governmental organisation, *PVO* private voluntary organisation

### Data analysis

The team analysed survey responses of conference participants using Microsoft Excel. Analysts imported interview transcripts and survey data into MAXQDA qualitative data analysis software (version 18) to facilitate coding open-ended questions and transcripts. Descriptive statistics (frequencies) were generated for demographic variables (e.g. country, type of work, years of experience) and variables of knowledge use and sharing (e.g. with whom knowledge was shared, types of use) to explore trends in characteristics among the study participants.

The study team applied qualitative data coding techniques [25] in analysing the transcripts and open-ended question data from the survey, using both deductive and inductive approaches [25]. Deductive coding primarily used a priori*-*defined domains from the validated TDF [16], ones identified in the literature concerning barriers and facilitators to evidence use, and concepts about evidence use and sharing behaviour from the survey. Researchers added a priori codes above those derived from the TDF in order to better capture the influence of factors relevant to the KB role but not explicit in the TDF such as evidence characteristics (e.g. Timely Relevance) and KB activities (e.g. Interpersonal Sharing). As coding progressed, researchers found that the added codes helped capture the relation between internal and external factors in the KB thought processes. Subsequent cycles of coding resulted in additional inductive codes. The team created a reference codebook of codes, definitions and examples to aid consistency in coding.

TN served as the primary coder throughout the study. To assess coding reliability, two authors (TN and DR) coded two transcripts purposively selected to have exceptionally deep content (i.e. extensive details in answers) and representing multiple types of respondent work and organisations. After provisional coding, points of disagreement were discussed and changes were made to the codebook.

Coding of the remaining interview transcripts took place in two cycles [25]. In the first cycle, all transcripts were coded using the amended codebook. The second cycle included revising the codebook as needed to reflect identified patterns, including further clustering and categorisation of codes into internal and external influences. Memo writing supported tracking rationales for code changes.

Analysis of coded segments included identifying the most relevant influences on the KB decision process. The criteria for relevance included rich descriptions (suggesting the importance of the topic to the respondent as an influential factor) and frequency across respondents. Similar tests for estimating relevance have been used in other TDF studies [11, 26]. Researchers used MAXQDA features in multiple ways to categorise and relatively rank influences. The codes ‘Barrier’ and ‘Facilitator’ were created and applied in tandem with construct codes to enable comparisons of which constructs appeared more as barriers or facilitators. Grouping codes as internal versus external influences similarly enabled comparisons. Finally, the MAXQDA feature of displaying the number of coded segment per code contributed to a relative ranking of codes and groupings, in combination with review of rich descriptions.

Continued analysis consisted of grouping and comparing the importance of internal facilitators, internal barriers, external facilitators and external barriers across data sources and respondent characteristics. Thematic analysis [27] yielded two types of insights, namely (1) themes of influences on the KB decision process, from the respondent perspective, and (2) themes of rationales for changing and supplementing TDF codes, from the researcher perspective, which are discussed in this paper.

#### Rigor and validity methods

The study design addressed rigor through multiple methods documented in the literature [28]. For quantitative data, researchers used validated survey and interview instruments. The interview sample was selected from the pool of survey respondents in line with a sequential study design. For qualitative data, multiple data sources – survey text responses, interviews and conference documents – were triangulated to assess consistency in findings and gain a deeper understanding of the study context. In addition to exploring themes of knowledge use and sharing, researchers also explored disconfirming evidence, i.e. accounts of not using or sharing knowledge from the conference. The study procedures also aimed for validity and included training interviewers according to developed standard operating procedures to ensure consistency, maintaining an audit trail of documentation such as interview logs, transcription verification records, researcher memos to record and interim analysis reports for expert reviewers.

## Results

Most respondents in the study (92.8%; *n* = 292) indicated that they shared knowledge that they gained from the conference. When asked to identify with whom sharing took place, the majority of responses indicated recipients the respondents knew, such as members of their organisation (85.0%; *n* = 267) or their professional network (51.7%; *n* = 267). Most responses about what they shared focused on sharing expert opinion (62.2%; *n* = 267) or experience from another participant (60.3%; *n* = 267). Respondents shared mostly by passing along conference materials (73.0%; *n* = 267) or mentioning knowledge in communications done in-person, by phone or through e-mail (71.2%; *n* = 267). The most common types of use were designing health projects or programmes (54.7%; *n* = 256) or improving healthcare service quality (50.0%; *n* = 256).

Qualitative data also indicated that conference participants did not act on evidence from the conference in isolation, but instead were sharing evidence with other health system actors to facilitate understanding and inform policy and practice, in line with characteristics of the KB role. The ways the facilitation occurred reflected the KB’s characteristics, context and thought processes about the evidence. For example, a researcher respondent described applying evidence about newborns to advocate with decision-makers to change how stillbirths are measured, whilst a healthcare provider respondent worked with colleagues to improve skills for newborn resuscitation.

Adequately exploring thoughts about evidence that KBs expressed required iteratively refining codes and definitions during analysis to address the scope of influences beyond those in the TDF. In particular, codes needed to be added or adapted to account for how KBs’ internal reflections on external factors influenced their actions in selecting knowledge to share and use and the decisions they made during the process.

### Changes to the TDF

All TDF domains were applied during interview transcript coding, whilst most were applied during coding of survey open-text responses. Most TDF domains represented internal influences such as beliefs and motives, whilst non-TDF codes represented a mixture of internal and external factors. The iterative coding process led to modification of the labels or definitions of four TDF domains – Knowledge, Skills, Intentions, and Environmental Context and Resources. Multiple reasons for modifying TDF domains surfaced during the coding process. In assessing the relevance of codes to the KB accounts based on rich descriptions and frequency across respondents, researchers decided that some relevant codes represented concepts that were important enough to respondents to merit adapting the TDF for the current study. For example, nuanced differences in how the domain Environmental Context and Resources was relevant to KBs appeared across accounts. In some cases, the environmental context referred to the KB’s own organisation and how it operated, with implications for brokering use of evidence with local partners, as with this example:

“*We* [my organisation] *are working with six hospitals, and we are applying social accountability tools to improve newborn health and also we include on what kind of facilities and equipment they are using, and how they are managing the equipment in their hospitals.*” (Health/Medical Service Delivery, Local non-governmental organisation (NGO), Southern Asia)

Environmental context also appeared as important to KBs regarding the structure of the health system in their country and how it related to implementation of evidence-based practices, as shown in this quote:

“*We are struggling in* [our country] *to establish the midwifery-led care, so midwife can link the community and the health facilities, and if the babies and mothers need the high quality or entrance care they can divert the mothers and the newborns in the tertiary health facilities level.*” (Teaching/Training, Government/Ministry, Southern Asia)

Other modifications were based on researcher reflection that several of the definitions seemed better suited to exploring behaviour related to one practice in a specific setting, rather than a broad array of knowledge applied in diverse settings that was characteristic of the current study. The rationale for the TDF changes aligns with published guidance to focus application of a given framework to the scope of a study [29] and to propose hypothetical domains and constructs as part of a validity process [30]. See Table 2 for TDF domains and definitions, and how they were changed in this study. The table also notes for each domain whether it was determined to be an internal or external influence and a key barrier or facilitator based on analysis.

**Table 2:** Changes to TDF domains and definitions during coding

Original TDF label	Original TDF definition [16]	Revised TDF label	Revised TDF definition	Internal or external influence	Key facilitator (+) or barrier (−)
1. Knowledge	An awareness of the existence of something	Knowledge and Learning	An awareness of the existence of something; Process of acquiring knowledge	Internal	+ −
2. Skills	An ability or proficiency acquired through practice	Unchanged	An ability or proficiency acquired through practice; includes individual capability for critically appraising research evidence and determining implications for action and costs; includes capability for adapting evidence for use or sharing in a local context or for current purposes	Internal	N/A
3. Social/professional role and identity	A coherent set of behaviors and displayed personal qualities of an individual in a social or work setting	Unchanged	Unchanged	Internal	+ −
4. Beliefs about capabilities	Acceptance of the truth, reality or validity about an ability, talent or facility that a person can put to constructive use	Unchanged	Unchanged	Internal	N/A
5. Optimism	The confidence that things will happen for the best or that desired goals will be attained	Unchanged	Unchanged	Internal	N/A
6. Belief about consequences	Acceptance of the truth, reality or validity about outcomes of a behavior in a given situation	Unchanged	Unchanged	Internal	+
7. Reinforcement	Increasing the probability of a response by arranging a dependent relationship, or contingency, between the response and a given stimulus	Unchanged	Unchanged	Internal	N/A
8. Intentions	A conscious decision to perform a behavior or a resolve to act in a certain way	Unchanged	A conscious decision or plan to use or share knowledge; displaying initiative in evidence use or sharing	Internal	+
9. Goals	Mental representation of outcomes or end states that an individual wants to achieve	Unchanged	Unchanged	Internal	N/A
10. Memory, attention and decision processes	The ability to retain information, focus selectively on aspects of the environment and choose between two or more alternatives	Unchanged	Unchanged	Internal	–
11. Environmental context and resources	Any circumstances of a person’s situation or environment that discourages or encourages the development of skills and abilities, independence, social competence and adaptive behaviour	a. Environmental context – Own Organisation and Setting b. Environmental context – Country or Health System c. Resource Availability	The domain was split into three sub-domains, as follows: a. Aspects of a person’s organisation or setting that influence behaviour or actions regarding evidence use or sharing b. Country or health system characteristics that influence behaviour related to evidence use or sharing c. Availability of financial and other types of resources (e.g. human, supplies) for using or sharing evidence; includes the financial resources needed to incorporate the evidence in health practice	External	–
12. Social influences	Those interpersonal processes that can cause individuals to change their thoughts, feelings or behaviours	Unchanged	Unchanged	External	N/A
13. Emotion	A complex reaction pattern, involving experiential, behavioural and physiological elements, by which the individual attempts to deal with a personally significant matter or event	Unchanged	Unchanged	Internal	N/A
14. Behaviour Regulation	Anything aimed at managing or changing objectively observed or measured actions	Unchanged	Unchanged	Internal	N/A

### Supplemental codes

Table 3 presents the most used non-TDF codes that were added deductively and inductively to capture a range of internal and external barriers and facilitators. Most supplemental codes aided in identifying influences relevant to complex environments, such as those of LMICs, and the unique nature of the KB experience. For example, the supplemental code Multi-Country Importance was useful across KB accounts in different ways from TDF constructs and was possibly more relevant to complex settings. The code relates to the view that critical health issues and implementation evidence are more important when relevant to multiple countries. This view expressed by multiple KBs appeared more often in mention of low-resource settings; however, one interesting exception stressed a common healthcare issue across countries with dissimilar economic characteristics, as shown in this quote:

“*It is quite comparative, that I have, for example, the newborn screening program which is not available for other African or Asian countries, and in spite of that I have the problem of newborn screening because it is not democratically distributed in the whole country, for the wealthy countries as well.*” (Health/Medical Service Delivery, Private Sector, Western Asia)

The table includes a short name and definition, and notes of whether the code represented an internal or external influence and was determined to be a key facilitator or barrier.

**Table 3:** Most used supplemental codes

Code label	Definition	Internal or external influence	Key facilitator (+) or barrier (−)
Accessibility	Ease or difficulty of obtaining evidence when and where it is desired and in the format desired	External	–
Decision-Making Culture	Collective characteristics and knowledge of a group of people that influence individual decision-making	External	–
Interpersonal Sharing	Interpersonal communication among research producers and consumers or stakeholders as part of a relationship that includes discussion of research evidence	Internal	+ −
Knowledge Presentation	Suitability of presentation of evidence, language for intended audience, synthesised evidence and knowledge products	External	+ −
Local Applicability	Belief about the relevance of evidence from a global source or other country to a local setting, whether now, in the past or in the future	External	+
Multi-Country Importance	Public health problems, evidence or interventions that are important to multiple countries or globally	External	+
Opportunity Availability	Availability of time or opportunity in the course of professional duties to use or share knowledge	Internal	–
Timely Relevance	Belief that research topic is relevant to current or near-term work or organisational objectives	External	+
Usefulness	Extent to which knowledge can be used for a practical purpose or in several ways	External	+

### Themes of code changes

The issues with adequately capturing influences on KBs that led to code changes can be seen as falling within four major themes.

#### Influences from beyond the organisation

The most essential change needed to the TDF – and requiring addition of non-TDF codes –concerned influences originating beyond the respondent’s organisation or immediate environment. In the complex arena of global health, these influences included ones within the country (such as policy environment, culture and health system), from other countries, and from global organisations such as WHO.

The abundance and breadth of data applicable to the TDF domain Environmental Context and Resources led the team to subdivide the domain into three areas of influence, namely (1) aspects of the organisation or setting, (2) characteristics of a country or health system, and (3) availability of financial, human or other resources. Each distinction aided understanding of the data and has implications for possible interventions to aid evidence use and sharing. For example, the ability to note country or health system contextual factors surfaced issues with the governing environment that impeded evidence use.

“*There is a focus on integrations of maternal and newborn health in that conference* [but] *in our…country at a central level there is no proper coordination between two divisions, maternal health and child health.*” (Programme Development/Management/Implementation, NGO/Private Voluntary Organisation (PVO), Asia)

As the conference knowledge was intended for use in resource-limited environments, the study team also found it useful to be able to determine the extent to which the availability of resources influenced evidence use.

“*So you pass the information to a lower cadre that should do the work, but very often they don’t have the stethoscope and…to check the blood pressure, or they don’t have the urine dipstick to check for the proteinuria.*” (Research/Evaluation, NGO/PVO, Africa)

The significance of international influences became evident during the coding process and led to the addition of several non-TDF codes, namely Multi-Country Importance, Comparable Setting and Success, and International Relationships. These codes helped fill a gap in capturing the value that KBs placed on evidence generated or applied in other LMICs that had similar characteristics (e.g. economic, cultural).

“*...whenever we propose some kind of suggestion…public health officials…ask us…have you any good success stories, where from you collected this kind of idea. So we suggest to them that… we come to know about such kind of practices being used in other countries, and their economic situation and their electricity situation is the same as ours. So we can do such kind of* [idea] *easily and these are easily applicable...*” (Health/Medical Service Delivery, NGO/PVO, Asia)

#### Knowledge selection as a process

In other studies using the TDF, the knowledge linked to behaviour has already been selected and a specific action identified (i.e. a ‘best practice’, such as washing hands to prevent infection). Individuals apart from those targeted in the studies have decided that the knowledge is relevant to address a timely problem relevant to a local context. For this study, labels and definitions needed to be adapted to reflect the process KBs undergo in identifying and deciding what to do (if anything) about knowledge to which they had been exposed. For example, the domain Knowledge relates to whether someone has awareness of information to the extent that they could take a proscribed action. With KBs, however, perceiving that they had learned something new appeared to be a facilitating precursor to focusing attention on and acting upon the knowledge.

“*It was my first time to attend the…international conference. To me it was like I didn’t know that maybe what I was looking at... I didn’t know that it was very, very important, and it’s not only in* [my country]” (Teaching/Training, Academic/Research Institution, Africa)

For this reason, the team expanded the domain and definition to include the process of acquiring knowledge (Knowledge and Learning).

Similarly, the domain Skills was clearly relevant to KB descriptions of learning a specific clinical skill such as newborn resuscitation; however, it omitted a crucial aspect of the knowledge selection process, namely the ability to critically appraise knowledge as scientifically or otherwise valid and contextually relevant.

“*Okay, one of the major reasons* [I shared the knowledge about newborn resuscitation technique] *is that… if you go on a deep analysis of some of the causes* [of high] *neonatal mortality rates, most of these, they are somehow preventable deaths if we apply correct skills.*” (Health/Medical Service Delivery, Government/Ministry, Sub-Saharan Africa)

Expanding the definition of Skills enabled the study team to capture both narrowly defined clinical skills (e.g. resuscitation technique) and broader reasoning skills (e.g. suitability for addressing newborn mortality) involved in the KB process.

Another need for extending domain definitions related to the later stages of the knowledge selection process, during which KBs considered the possibilities of future action based on knowledge. Given that the KBs in this study also mentioned seeing themselves as responsible for applying knowledge, the team wanted to capture KB traits of initiative and action-orientation as facilitators in the process. To address this need, the team expanded the definition of the domain Intentions from planning on adhering to specific guidelines to a broader willingness to take the initiative on knowledge action.

“*Working with the nurses, we tried to share with them the importance of making sure that they provide quality services to the antenatal clinic. This is one of the lessons I learned at the conference. I try to make sure that at any time I speak to nurses and midwives, I emphasize the issue of the content of care they give to their client.*” (Research/Evaluation, NGO/PVO, Africa)

#### Access and packaging of knowledge

With many of the studies using the TDF or relating to KBs taking place in higher-income countries, access to knowledge in a needed format and language is seldom depicted as a significant issue. With the conference knowledge being intended for use in resource-limited settings, the study team identified the need to capture external influences related to knowledge access in LMICs. These influences included access to knowledge synthesis products and electronic resources (in lieu of print resources). To capture these concepts, the team added the codes Accessibility and Knowledge Presentation, which appeared relevant as both a facilitator and barrier. In one example, the respondent mentioned the challenge in arranging for a flow of information from urban to rural settings for local use.

“*I often have information for me because most of the time I live in the state capital. I don’t go to villages or rural area, where people that need the information live. So when you come back* [from a conference] *you have to identify people to actually go to those villages or rural areas who will be able to pass this information down the line…to people that need the information to change their life.*” (Research/Evaluation, NGO/PVO, Africa)

KBs also mentioned the need for them to filter information from the conference that had been packaged for an audience working globally rather than locally.

#### Fit for use

In many studies using the TDF, the evidence in question has been previously determined to be a good fit for the context. With the global nature of the conference, the evidence presented could potentially be shared and used in settings worldwide. KBs described their thought processes in identifying potentially actionable evidence and assessing whether and how it could be adapted for local use. In order to capture this reasoning, the study team added codes for Adaptability of Evidence and Local Applicability.

*“Because it is a cultural practice* [in our country] *that people apply something on the umbilical cord of a newborn child. And if you provide them with the something which is safe* [chlorhexidine] *and which will prevent sepsis… so that’s why it was a decision that instead of one day it should be a seven day application. So we adapted it to our cultural practices*.” (Policy-making, Government/Ministry, Asia)

### Relevance of TDF versus non-TDF codes

**Fig. 2:**
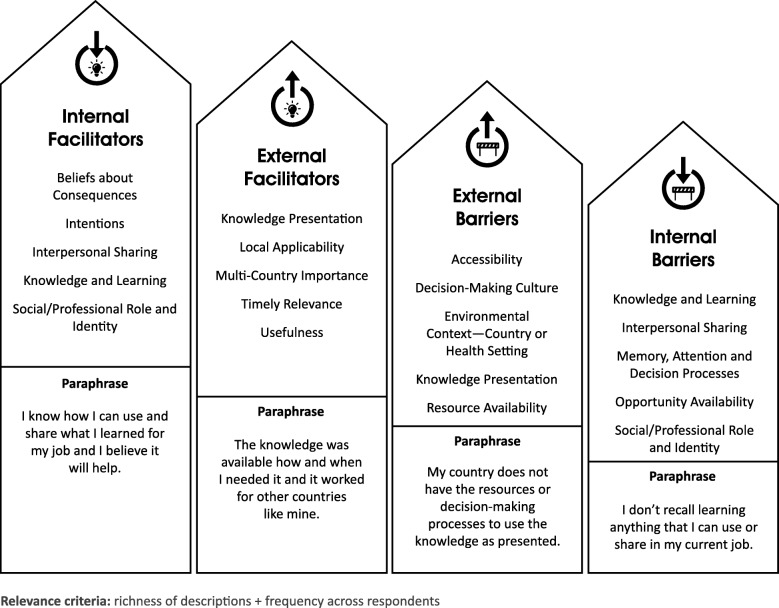
Most relevant facilitators and barriers to knowledge sharing and use, in order of declining importance (left to right). Internal facilitators appeared most influential on knowledge brokers

Overall, codes categorised as internal facilitators appeared most influential on respondents, followed by external facilitators, external barriers and, finally, internal barriers. Figure 2 shows the most relevant codes by category. Researchers arrived at relative rankings through a review of frequencies of coded segments and code groupings in MAXQDA and review of rich descriptions to assess KB views of importance. Using this combination of approaches, researchers relatively ranked the top five codes in each category pair (barrier/facilitator, internal/external).

TDF-based codes and inductively derived codes were equally useful in identifying relevant internal facilitators and barriers. Inductively and deductively derived non-TDF codes were most helpful in identifying relevant external facilitators and barriers, although Environmental Context and Resources is a concept that appears both in the TDF and other literature.

The top relevant domains and relative importance of internal versus external influences appeared to be mostly consistent across respondent characteristics such as type of work, type of organisation and region. Slight differences in internal versus external influences appeared when comparing respondents by type of work. For example, for respondents working in Health/Service Delivery, external barriers were more influential than for respondents working in other types of work. One nuanced difference among the rich descriptions, though not affecting estimations of relevance, concerned the Emotion domain. Expressions of passion for improving health outcomes in an LMIC appeared almost exclusively with respondents who both worked and lived in an LMIC country. Respondents based in one country, but working on activities in other countries, did not express passion to the same extent.

## Discussion

Using the TDF provided a starting point in this study to identify the internal barriers and facilitators to evidence sharing and use described by KBs; however, its use was somewhat challenging and required adaptation to address broad external factors and adequately explain knowledge brokering behaviour.

### Using TDF to explore the interpersonal communication aspect of knowledge brokering

Analysis of qualitative data using TDF-derived codes helped identify factors that relate to effective knowledge brokering, which may be because of the TDF focus on human behaviour and its underpinnings in psychological theory. Interpersonal communication has been identified in the literature as an essential element in knowledge brokering and in advocating use of evidence in public health decision-making [13, 31–34]. The need for KBs to build relationships of trust with health decision-makers [33] requires a strong foundation of interpersonal skills. Use of the TDF may have helped authors identify internal factors influencing evidence sharing and use more so than using an inductive approach alone.

The TDF was useful in exploring KB characteristics as well. Studies have examined individual aspects of KBs, and there have been calls for additional research on desirable KB personal attributes [31]. Positive traits for KBs identified in the literature include professional competencies [13, 35, 36], experiential knowledge [13], interactive skills [32] and personal disposition (e.g. a strong commitment to improving health outcomes in their country, action orientation) [13]. These attributes exhibited themselves in the current study through the KB descriptions of how they shared and used evidence in their professional roles, demonstrating confidence in their professional competence, experience, interpersonal skills and vision for improved health outcomes. The findings suggest that KBs in the study may fit the profile of a type of KB referred to in the literature as a ‘knowledge mobiliser’ [37] who can drive change and operationalise evidence. Further research based on the findings may contribute to development of an assessment tool to identify KBs who are also mobilisers.

Most findings of influences on evidence use surfaced by the TDF agree with ones mentioned in the literature, whilst a few disagreed. The high relevance of Belief About Consequences coincides with findings showing that healthcare professionals tend to act on knowledge if they believe it will have positive consequences for the care of patients (or not act upon it in the case of anticipated negative outcomes) [11, 38–40]. The strong influence on KBs of the anticipated health outcomes resulting from evidence use may link to the fact that many KBs in the study had hybrid professional roles, including responsibility for applying knowledge (such as through healthcare service delivery) as well as knowledge brokering. Social and Professional Role [11] and Environmental Context [39] also appear as highly relevant TDF domains in other studies, as in the current research, suggesting influences that may be generalisable across types of roles and settings in the health field. On the other hand, Social Influence appears highly relevant in multiple studies [39, 40], but not in the current study. The difference in relevance may be because the diversity of evidence, job roles and settings in the present research did not surface reflections on social influence the same way that a study focusing on one clinical behaviour and one or few healthcare cadres might. An alternative explanation may be that KBs in the study did not mention social influences on their professional decisions out of a social desirability bias.

### Challenges with applying the TDF

The authors experienced several difficulties with applying the TDF, some of which the literature also reports. The time-consuming aspect of utilising the TDF posed a problem that has been mentioned in other research [10, 29, 41], though the extent to which using the TDF added to the time that qualitative data analysis typically takes is not known. Identifying distinctions between domains also posed a challenge noted in other studies [26]. The presence of the same constructs in multiple TDF domains added to the challenge. For example, Professional Confidence is a construct included in two TDF domains – Social/Professional Role and Identity and Beliefs about Capabilities. The authors in this study addressed the challenge by iteratively revising their qualitative codebook, adding to the exemplars and inclusion and exclusion criteria, which added to the analysis time.

Other challenges concerned how the study design incorporates use of the TDF. The current study used the TDF retrospectively on existing data, instead of prospectively for instrument design and analysis. While the TDF has been successfully used retrospectively (e.g. systematic review of implementation interventions [39]), such an approach has a risk of missing barriers and other factors that might surface during instrument design [26, 41]. Retrospective use in this study also involved applying the TDF to a broadly defined behaviour (knowledge sharing and use), as opposed to one specific practice, which may have been why some TDF domains were underrepresented (e.g. Reinforcement). If the study had prospectively focused on one behavior and professional role within the realm of knowledge brokering, such as academic KBs interacting with policy-makers, these underrepresented domains might have surfaced. Issues also arose from applying TDF to the topic of global health. Contextual factors are so complex in LMICs that the TDF domain Environmental Context and Resources needed to be expanded and adapted to capture the critical implications of knowledge exchange between countries.

Finally, the authors experienced challenges in distinguishing between internal and external factors and, similarly, between barriers and facilitators. For example, the way that respondents reflect on external factors could be said to be determined by their internal attributes. In one case, a public health decision-maker displayed an internal skill in crafting messaging about a health practice in response to an external factor, that is, resistance of local healthcare providers. Similarly, a respondent’s view of whether an external factor was a barrier or facilitator may have been determined by internal factors. For example, in a case of a healthcare service provider describing cultural practices for newborn cord stump care, the provider described the practices as an opportunity to substitute an evidence-based approach in a culturally acceptable way rather than a barrier.

Use of the TDF offered a starting point for exploring KB decision processes, but might not be as useful if study aims focus on implementation of a particular intervention, for which there are other frameworks. Notable among these implementation frameworks are the Tailored Implementation for Chronic Diseases – Determinants of Practice Checklist [17], Promoting Action on Research Implementation in Health Services [42], and the Consolidated Framework for Implementation Research [43]; however, these frameworks do not share the same focus as the TDF. They are primarily concerned about implementation of interventions rather than the earlier steps of engaging with evidence to prioritise and adopt interventions, and they focus on organisational aspects rather than individual ones. Further research would be needed to determine if the supplemental codes created for the current study, and particularly Multi-Country Importance, would provide a useful addition to these popular merged frameworks.

### Limitations of the study

The study had limitations related to sampling, respondent bias, study timeline and TDF use. First, self-selection of study participants may have led to over-reporting of facilitators and under-reporting of barriers due to social desirability bias – volunteers for the study may have wanted to show appreciation for being included in the conference, particularly those who received sponsorship to attend. In turn, over-reporting and under-reporting may have influenced estimations of which TDF domains were most relevant to KBs. Insufficient samples of respondents for key demographic characteristics such as region and type of work limited the ability of the authors to identify how domain relevance may have varied by strata. Second, the length of time between the conference and interviews (10–14 months) may have introduced a recall bias. The extended timeline also made it infeasible to obtain comments from study participants about the findings. Finally, the authors’ adaptation of the TDF meant that they were no longer working with a validated version of the TDF, and the changes were not validated.

## Implications and next steps

Additional research should be conducted to build on the theoretical contributions of the TDF to explore internal and external factors influencing evidence sharing and use in LMICs. Additionally, integration of TDF with commonly used implementation frameworks should be explored for interventions in LMICs that have knowledge brokering or dissemination as a critical component. Use of the TDF in building KB capacity in key influential areas, such as the interpersonal skills involved in knowledge brokering, should also be explored.

## Conclusions

Theories of individual behaviour such as those in the TDF can help understand when, where and for whom knowledge brokering is effective in increasing evidence-informed health policy and practice in LMICs. Understanding how KBs in LMICs reflect on evidence and interact with their environment has potential for improving global dissemination efforts and LMIC-to-LMIC exchange of evidence and implementation approaches.

Box 1 TDF domains [16] 1. Knowledge

2. Skills

3. Social/professional role and identity

4. Beliefs about capabilities

5. Optimism

6. Beliefs about consequences

7. Reinforcement

8. Intentions

9. Goals

10. Memory, attention and decision processes

11. Environmental context and resources

12. Social influences

13. Emotion

14. Behavioural regulation

## Additional files

Additional file 1: Survey questions. (PDF 90 kb)

Additional file 2: Interview script. (PDF 69 kb)

## Data Availability

The datasets used and/or analysed during the current study are available from the corresponding author on reasonable request.

## Abbreviations

KB: knowledge broker
KT: knowledge translation
LMICs: low- and middle-income countries
NGO: non-governmental organisation
PVO: private voluntary organisation
TDF: Theoretical Domains Framework
