# Supplementary material for: Applying the Theoretical Domains Framework to understand knowledge broker decisions in selecting evidence for knowledge translation in low- and middle-income countries
Source: Health Res Policy Syst. 2019 Jun 11;17:60. doi: 10.1186/s12961-019-0463-9 (PMC6560763; doi:10.1186/s12961-019-0463-9)
Supplement: Supplementary file 1 — Survey questions. (PDF 90 kb) [file 12961_2019_463_MOESM1_ESM.pdf]

Global Maternal Newborn Health Conference 2015
Knowledge Sharing and Use Participant Survey

Note: Required questions are noted with an asterisk (*). Question type is included in parentheses after each question.

Part 1 – Responder Information

1. How did you attend the 2015 Global Maternal and Newborn Health Conference?* (select one)
    a. Online
    b. In-person
    c. I did not attend the conference

2. In which country are you primarily based?* (select one)

3. Please select the category that best describes your organization type.* (select one)
    a. Academic/research institution
    b. Donor
    c. Government/ministry
    d. Medical/health organization
    e. NGO/PVO (local and international)
    f. Private sector (for-profit)
    g. UN system
    h. Other (please specify)

4. Please select the category that best describes your work.* (select one)
    a. Advocacy
    b. Health communication
    c. Health/medical service delivery
    d. Policymaking
    e. Program development/management/implementation
    f. Research/evaluation
    g. Teaching/training
    h. Student
    i. Other

5. Please select the number of years that you have been employed in your current field.* (select one)
    a. 0-5 years
    b. 6-10 years
    c. 11-15 years
    d. 16 or more years
6. Did you attend the post-conference meeting for staff of the Saving Newborn Lives Program and Maternal and Child Survival Program? (Yes, No, Do not know)

7. Did you have an abstract accepted for the conference? (Yes, No, Do not know)

8. What were the top three reasons that you attended the conference? Please rank three choices, with 1 being the most important and 3 being the least important. Select only three.
    a. I had an abstract or presentation accepted
    b. I served as a member of a country delegation
    c. I wanted to learn best practices
    d. I wanted to exchange ideas with other participants about what works in their countries
    e. I wanted to develop hands-on skills, such as newborn resuscitation
    f. I wanted to learn about new evidence from global experts
    g. My organization required that I attend
    h. Other (please specify)

Part 2 – Knowledge Sharing and Use

9. Have you shared information or knowledge from the conference? (Yes, No, Do not know)

10. With whom have you shared information or knowledge gained from the conference? Select all that apply.* (select multiple)
    a. Members of my organization
    b. Communities of practice or working groups
    c. Clients or beneficiaries of my work
    d. Professionals I know in other organizations
    e. Online discussion groups
    f. Audiences of presentations
    g. Readers of my publications or other writings
    h. Students
    i. Connections through social media
    j. I have not shared information or knowledge from this conference
    k. Other (please specify)

11. Which types of information or knowledge from the conference have you shared? Select all that apply.* (select multiple)
    a. Expert opinion
    b. Experience from another participant
    c. Country-specific information
    d. Clinical or scientific information
    e. Information about a journal article or other publication
    f. I have not shared information or knowledge from this conference
    g. Other (please specify)

12. How have you shared information or knowledge gained from the conference? Select all that apply.* (select multiple)
    a. Shared conference materials (e.g., handouts, PowerPoint files) with others
    b. Mentioned in communication (in-person, phone, e-mail)
    c. Included or cited information in a publication, presentation, or proposal
    d. I have not shared information or knowledge from this conference

e. Other (please specify)

13. What influenced your decision to share information or knowledge from the conference? Select all that apply.* (select multiple)
    a. Wanted to share information that I thought would be useful to a co-worker or colleague
    b. Wanted to share information that I thought would be useful to others in my field
    c. Wanted to share information that was previously unknown to me
    d. Hoped it would help improve a global health program
    e. Thought it would lead to improved service delivery
    f. I have not shared information or knowledge from the conference

14. How many times do you estimate you have shared information or knowledge from the conference?* (select one)
    a. 10 or more times
    b. 7-9 times
    c. 4-6 times
    d. 1-3 times
    e. I have not shared information or knowledge from this conference

15. If you have not shared information or knowledge from the conference, why not? Select all that apply.* (select multiple)
    a. Not enough time to share
    b. Did not identify need to share information or knowledge
    c. Language of information or knowledge was a barrier
    d. Other (please specify)

16. Have you used information or knowledge from the conference? (Yes, No, Do not know)

17. Have you used information or knowledge that you have gained from the conference to do any of the following? Select all that apply.* (select multiple)
    a. To design projects or programs
    b. To advocate for policy change
    c. To develop training programs or design educational materials
    d. To improve service quality
    e. To write funding proposals, reports, articles, or research papers
    f. I have not used information or knowledge gained from this conference
    g. Other (please specify)

18. Please give an example of how you have used information or knowledge from this conference, if applicable. (open text)

19. Thinking back on how you have used information or knowledge gained from the conference, please mark the extent of knowledge use resulting from each conference activity according to the scale below. Select one choice in each row.

	4<br>Extensive	3	2	1	0<br>Did not attend

	Use of knowledge later	Some use of knowledge later	Little use of knowledge later	No use of knowledge later	
Plenaries (including the opening and closing ceremonies)					
Concurrent sessions					
Poster sessions					
Receptions and tea/coffee breaks (as networking opportunities, discussions with other participants)					
Marketplace of ideas					
Skills demonstrations					
Outside meetings					

Part 3 – Survey Use

20. If you have not used information or knowledge from the conference, why not? Select all that apply.
    a. Not enough time to use
    b. Do not have the authority
    c. Health system constraints
    d. Not relevant to my type of work
    e. Not practical enough to use
    f. Language of the information or knowledge was a barrier
    g. Other (please specify)

21. Please feel free to add any additional comments you have about sharing and using information and knowledge from the conference.

22. May we contact you for a 30-minute interview to hear more about your experience using and sharing information and knowledge from the conference? If so, please provide your name and e-mail address below. (open entry)

    a. Name
    b. E-mail Address

23. Please re-enter your e-mail address.
