# Supplementary material for: Applying the Theoretical Domains Framework to understand knowledge broker decisions in selecting evidence for knowledge translation in low- and middle-income countries
Source: Health Res Policy Syst. 2019 Jun 11;17:60. doi: 10.1186/s12961-019-0463-9 (PMC6560763; doi:10.1186/s12961-019-0463-9)
Supplement: Supplementary file 2 — Interview script. (PDF 69 kb) [file 12961_2019_463_MOESM2_ESM.pdf]

Global Maternal Newborn Health Conference 2015 Knowledge Use and Sharing Study:

In-Depth Interview Questions

Date:

Interviewer Initials: Interview ID: Interview Language (if not English):

1. How did you attend the 2015 Global Maternal Newborn Health Conference? Ways to participate would be in-person or online, or you could say that you did not attend the conference.

2. In which country are you based?

3. FOR ONLINE PARTICIPANTS: Please describe your online participation experience.

4. Have you used information or knowledge you gained through the 2015 Global Maternal Newborn Health Conference in your work?
   __Yes __No

5. If no, would you talk about reasons for not using the knowledge?

6. If yes, I would like for you to think of the times you used information from the conference. Please talk about:
   a. what the information or knowledge was
   b. how you used it in your work
   c. whether your use of the information or knowledge affected healthcare practice or policy (for example, provider performance standards, national service delivery guidelines or policy)

7. From which conference activities did you most gain information or knowledge that you used in your work? The conference activities included:
   a. Plenaries (including the opening and closing ceremonies)
   b. Concurrent sessions
   c. Poster sessions
   d. Receptions and tea/coffee breaks (networking, discussions with other participants)
   e. Marketplace of ideas

    f. Skills demonstrations
    g. Outside meetings

8. Please think about the conference activities from which you gained the most information or knowledge. Why do you think you gained more knowledge from those activities over other activities?

9. Have you ever shared information or knowledge from the conference with others?
   __Yes __No

10. If no, would you please talk about reasons for not sharing knowledge?

11. If you have shared information, would you tell me more details? For example, would you tell me about:
    a. what type of information you shared,
    b. with whom you shared it, and
    c. how you shared it (for example, by e-mail or in-person).

12. If you shared information or knowledge from the conference, please tell me about your reasons for sharing.

13. Have you ever experienced barriers to using or sharing information or knowledge from the conference? __Yes __ No

14. If you have experienced barriers, please tell me about the barriers you have experienced.

15. This is the end of the interview. Is there anything else you like to tell me about the 2015 Global Maternal Newborn Health Conference – any other comments that you have?

Thank you for your time! I've enjoyed talking with you!
